# Clinical Application of Artificial Intelligence in Ultrasound Imaging for Oncology

**DOI:** 10.31662/jmaj.2024-0203

**Published:** 2024-09-27

**Authors:** Masaaki Komatsu, Naoki Teraya, Takashi Natsume, Naoaki Harada, Katsuji Takeda, Ryuji Hamamoto

**Affiliations:** 1 Cancer Translational Research Team, RIKEN Center for Advanced Intelligence Project, Tokyo, Japan; 2 Division of Medical AI Research and Development, National Cancer Center Research Institute, Tokyo, Japan; 3 Department of Obstetrics and Gynecology, Showa University School of Medicine, Tokyo, Japan; 4 Department of Gynecology, National Cancer Center Hospital, Tokyo, Japan; 5 HLPF Data Analytics Department, Fujitsu Ltd., Kawasaki, Japan; 6 Department of NCC Cancer Science, Biomedical Science and Engineering Track, Graduate School of Medical and Dental Sciences, Tokyo Medical and Dental University, Tokyo, Japan

**Keywords:** ultrasound imaging, oncology, artificial intelligence, SaMD, domain shift, explainability, acoustic shadow

## Abstract

Ultrasound (US) imaging is a widely used tool in oncology because of its noninvasiveness and real-time performance. However, its diagnostic accuracy can be limited by the skills of the examiner when performing manual scanning and by the presence of acoustic shadows that degrade image quality. Artificial intelligence (AI) technologies can support examiners in cancer screening and diagnosis by addressing these limitations. Here, we examine recent advances in AI research and development for US imaging in oncology. Breast cancer has been the most extensively studied cancer, with research predominantly focusing on tumor detection, differentiation between benign and malignant lesions, and prediction of lymph node metastasis. The American College of Radiology developed a medical imaging reporting and data system for various cancers that is often used to evaluate the accuracy of AI models. We will also explore the application of AI in clinical settings for US imaging in oncology. Despite progress, the number of approved AI-equipped software as medical devices for US imaging remains limited in Japan, the United States, and Europe. Practical issues that need to be addressed for clinical application include domain shifts, black boxes, and acoustic shadows. To address these issues, advances in image quality control, AI explainability, and preprocessing of acoustic shadows are essential.

## 1. Introduction

Advancements in artificial intelligence (AI) technologies have significantly influenced the medical field, particularly oncology, facilitating research and the development of personalized medicine ^(1), (2), (3), (4), (5)^. The Food and Drug Administration (FDA) listed AI- and machine learning (ML)-enabled medical devices marketed in the United States ^(6)^. By August 2024, the FDA had authorized 950 AI/ML-enabled medical devices, predominantly AI-equipped software as a medical device (AI-SaMD), for medical image diagnosis, such as computed tomography (CT), magnetic resonance imaging, and X-rays. In Japan, AI-based products undergo a different approval process than those in the United States and must be reviewed and approved by the Pharmaceuticals and Medical Devices Agency (PMDA). A dedicated SaMD review office was established within the PMDA in 2021. As of July 2024, PMDA-approved AI-SaMDs for medical imaging are mainly related to endoscopy and CT, and only 2 of 27 (7.4%) are for ultrasound (US) imaging (https://www.pmda.go.jp/PmdaSearch/kikiSearch/, accessed July 25, 2024).

US imaging is simple, noninvasive, and operates in real time compared with other medical imaging modalities. Advances in US technology, such as three- (3D) and four-dimensional (4D) imaging, portable devices, and point-of-care applications, have expanded their use across various medical fields, including oncology. However, the manual nature of US image acquisition can lead to variability in viewpoints, cross sections, and diagnostic accuracy, which are heavily dependent on the skills of the examiner. Acoustic shadows can degrade the image quality, which reduces diagnostic reliability. AI can address these issues and enhance image quality ^(7), (8), (9), (10), (11)^, but its limitations, such as reduced resolution, artifacts, and manual scanning, also make analysis difficult ^(12)^. Among the 950 FDA-approved devices, only 59 (6.2%) were related to US. Similarly, in Europe, only 10 (4.7%) of the 213 Conformité Européenne-marked AI-SaMDs are related to US (https://radiology.healthairegister.com/, accessed July 29, 2024) (Table 1) ^(13)^. Furthermore, to use AI-SaMD in clinical practice, ethical issues, such as patient consent and diagnostic responsibility, must be considered. This review explores the current progress and future directions of AI research and development in US imaging for oncology.

**Table 1.** List of AI-Based Ultrasound SaMDs Available in Europe (CE Marked).

No.	Product name (company)	Subspeciality	Description	Regulatory certification		On market since
				CE	FDA	
1	LVivo Toolbox - Cardiac (DiA Imaging Analysis)	Cardiac	An AI echocardiographic analysis solution that provides objective echocardiographic image analysis aimed at reducing the challenges associated with manually capturing and visually analyzing ultrasound images	Certified, Class IIa, MDD	510(k) cleared, Class II	2013
2	QVCAD (QView Medical)	Breast	An ANN-based system used as an adjunct to radiologists conducting breast screening exams for women who have negative mammogram results but dense breast tissue and therefore undergo 3D breast ultrasound (ABUS)	Certified, Class unknown	PMA approved, Class III	09-2016
3	AmCAD-UT^Ⓡ^ (AmCad BioMed)	Head and neck	An AI solution that quantifies and visualizes ultrasound image features to help physicians make informed decisions	Certified, Class IIa, MDD	510(k) cleared, Class II	2017
4	EchoGo Core (Ultromics)	Cardiac	An automated solution for cardiovascular findings to calculate ejection fraction, global longitudinal strain, and left ventricular volume	Certified, Class I, MDD	510(k) cleared, Class II	11-2019
5	EchoGo Pro (Ultromics)	Cardiac	An outcome-based AI system to predict coronary artery disease	Certified, Class I, MDD	510(k) cleared, Class II	03-2020
6	LVivo Bladder (DiA Imaging Analysis)	Bladder	A solution that transforms any on-premise ultrasound device into an AI-powered bladder scanner to accurately measure bladder capacity with a single click	Certified, Class IIa, MDD	510(k) cleared, Class II	06-2020
7	TRACE4OC (DeepTrace Technologies Srl)	Ovary	A decision support system based on radiomics and machine learning to predict the malignant risk of ovarian mass from transvaginal ultrasonography and serum CA-125 levels	Certified, Class I, MDD	-	05-2021
8	Koios DS (Koios Medical, Inc)	Breast and thyroid	An AI-based software application designed to help trained readers analyze breast and thyroid ultrasound images	Certified, Class IIb, MDR	510(k) cleared, Class II	12-2021
9	Ligence Heart (Ligence)	Cardiac	A platform that reads DICOM format data, recognizes image views and cardiac cycles, performs measurements, and generates reports based on the findings	Certified, Class IIa, MDR	-	04-2022
10	Us2.v1 (Us2.AI)	Cardiac	A machine learning-based solution that analyzes and interprets echocardiogram and ultrasound images of the heart to generate patient reports and is available on mobile, on-premises, and cloud-based platforms	Certified, Class IIb, MDR	510(k) cleared, Class II	06-2022

Abbreviations: AI, artificial intelligence; SaMD, software as a medical device; CE, Conformité Européenne; FDA, Food and Drug Administration; ANN, artificial neural network; MDD, medical device directive; MDR, medical device regulation.

## 2. Current AI Research and Development in US Imaging for Oncology

PubMed was searched for relevant studies published in the last 5 years using the following search strategy: (“ultrasound”) and (“cancer”) and (“artificial intelligence” or “machine learning” or “deep learning”) with an access date of July 25, 2024. Cancers are listed according to the number of published studies.

### 2.1. Breast cancer

Breast cancer remains the most extensively studied type in this field. Mammography (MG) is the primary screening tool, whereas US imaging is particularly useful for patients with dense breasts. The Breast Imaging (BI) Reporting and Data System (RADS) standardizes the interpretation and reporting of breast US. Yang et al. developed a deep learning (DL) model combining US and MG features that improved malignancy prediction in BI-RADS 4A breast lesions in patients with dense breasts ^(14)^. An AI system was developed to distinguish between the BI-RADS categories. The area under the receiver operating characteristic curve, sensitivity, and specificity were 0.95, 91.2%, and 90.7%, respectively. A comparison between the diagnostic accuracy of the 20 clinicians and the AI system showed that the AI system significantly outperformed the clinicians (*p* < 0.001) ^(15)^, leading to the PMDA approval of the AI-SaMD in 2024. Another retrospective reader study identifying breast cancer on US images revealed that the AI system achieved a higher area under the curve (AUC) than 10 board-certified breast radiologists (AI, 0.962; radiologists, 0.924), reducing false-positive rates by 37.3% and biopsy requests by 27.8% while maintaining sensitivity ^(16)^.

In a multimodal analysis incorporating US imaging and additional factors, a DL model was used to preoperatively differentiate between luminal and nonluminal early-stage breast cancers using US images and hematoxylin and eosin-stained biopsies ^(17)^. Accurate identification of axillary lymph node (ALN) involvement in patients with early-stage breast cancer is important for selecting appropriate treatment options. Combining DL radiomics from conventional US and shear wave elastography predicted ALN status between disease-free and metastatic axillae, with an AUC of 0.902. It also discriminated between low and high metastatic burdens of axillary disease, with an AUC of 0.905 ^(18)^. Guo et al. developed a multicenter DL radiomics model for predicting the risk of axillary nonsentinel lymph node involvement in primary breast cancer ^(19)^.

For triaging women with palpable breast lumps in low-resource settings, AI analyzed 758 masses in 300 women using portable US, categorizing them as benign, probably benign, suspicious, or malignant. This AI application to portable US images of breast masses accurately identified malignancies, with an AUC of 0.91 ^(20)^. Another AI system analyzed breast US images captured using a smartphone, delivering predictions in just 2 s and demonstrating 100% sensitivity and 97.5% specificity for malignant lesions ^(21)^. Given the shared imaging features between the thyroid and breast, a previous study evaluated an AI computer-assisted diagnosis software originally developed for thyroid nodules in breast lesions; however, the findings indicated that an organ-specific approach would improve diagnostic performance ^(22)^.

### 2.2. Thyroid cancer

Thyroid cancer is the second most extensively studied cancer in this field, partly because of its shared US imaging features with breast cancer. The American College of Radiology (ACR) developed the Thyroid Imaging (TI) RADS to categorize thyroid US. Several DL models have been developed to differentiate between benign and malignant calcified thyroid nodules ^(23), (24)^. For example, the Xception model achieved an AUC of 0.970 ^(25)^, and a multichannel Xception-based framework was proposed ^(26)^. The object detection model YOLOv3, combined with a nomogram, improved accuracy in the distinction between benign and malignant TI-RADS 4 thyroid nodules ^(27)^.

An ensemble DL model has shown superior accuracy compared with other thyroid classification methods and US radiologists ^(28)^. A multimodal model combining US and infrared thermal images ^(29)^ and advanced multimodal imaging using AI-optimized B-mode elastography and dynamic contrast US ^(30)^ have been proposed to aid diagnostic procedures. Radiomic features derived from US images and combined clinical data have proven effective in differentiating thyroid follicular carcinomas from adenomas ^(31)^.

A prospective multicenter study used US videos with multiscale, multiframe, and dual-direction DL models to preoperatively predict cervical lymph node metastasis in patients with papillary thyroid carcinoma ^(32)^. Furthermore, an integrated model incorporating DL, handcrafted radiomics, and clinical and US imaging features was used to diagnose lymph node metastasis in patients with papillary thyroid cancer ^(33)^.

### 2.3. Liver cancer

Liver cancer ranks among the top five causes of cancer-related deaths in 90 countries, with cases expected to rise over the next two decades ^(34)^. One study demonstrated the ability of AI in the detection of focal liver lesions in US videos ^(35)^. Comparing tracking algorithms from the OpenCV library for liver tumors in US videos, a correlation filter tracker with channel and spatial reliability and a modified tracker demonstrated over 70% Intersection over Union and >85% successful searches in real-time processing ^(36)^. Using a large-scale dataset of 50,063 US images and video frames from 11,468 patients, Xu et al. developed a fully automated AI pipeline that mimics the workflow of radiologists in the detection of liver masses and in diagnosing liver malignancy ^(37)^. The AI models outperformed human experts in distinguishing between four types of tumors (cysts, hemangiomas, hepatocellular carcinoma, and metastatic tumors) as well as their benign or malignant classification ^(38)^. Moreover, the segmentation of blood vessels in liver US images can be an automatic CT registration pipeline to guide laparoscopic liver resection ^(39)^.

### 2.4. Pancreatic cancer

Pancreatic cancer has the poorest prognosis, with a 5-year survival rate of 11% ^(40)^. The use of endoscopic US (EUS) for diagnosis and treatment has gained attention. EUS-guided procedures enable early cancer detection and minimally invasive palliative or antitumor treatment, improving the patients’ quality of life. YOLOv5 effectively differentiates pancreatic cancer from nonpancreatic cancer lesions in real time using EUS images ^(41)^. Automatic segmentation of pancreatic tumors using U-Net on contrast-enhanced EUS video images was associated with good concordance ^(42)^. A DL-based system using deep convolutional neural networks and random forest algorithms can facilitate pancreatic mass diagnosis and EUS-guided fine-needle aspiration in real time ^(43)^. Zhang et al. developed a system for EUS training and quality control using ResNet for image classification and U-Net++ for image segmentation, achieving 90.0% accuracy in classification and 0.770 and 0.813 dice in blood vessel and pancreas segmentation, respectively. These results are comparable with those obtained by experts ^(44)^. An ML model predicted the clinical diagnosis of chronic pseudotumoral pancreatitis, neuroendocrine tumors, and ductal adenocarcinoma with an AUC of 0.98 and overall accuracy of 98.3% ^(45)^.

### 2.5. Ovarian cancer

Ovarian cancer is the leading cause of gynecological cancer-related deaths worldwide; however, screening methods have not been established. Previous trials combining cancer antigen testing with transvaginal ultrasonography did not significantly reduce mortality ^(46)^. A DL radiomics model was developed to differentiate between benign, borderline, and malignant ovarian tumors using US imaging ^(47)^. US image analysis using an ensemble of deep neural networks predicted ovarian malignancy with diagnostic accuracy comparable with that of experts ^(48)^. A DL radiomics nomogram based on US imaging accurately predicted the malignancy risk of ovarian tumors, which is consistent with the guidelines for the ovarian adnexal RADS published by the ACR ^(49)^. OvcaFinder was developed as an interpretable model that combines US image-based DL predictions, ovarian adnexal RADS scores, and routine clinical variables ^(50)^. Gao et al. conducted a retrospective, multicenter study using a large dataset to develop a deep convolutional neural network model for the automated evaluation of US images, which significantly enhanced the accuracy and sensitivity of diagnosis compared with radiologists alone. Further prospective studies or randomized clinical trials are required to confirm these findings ^(51)^.

### 2.6. Other cancers

Several AI studies using US imaging have been conducted for various cancers, beyond those mentioned. One study investigated a 3D DL model for segmenting tongue carcinomas using 3D US volumes during surgery ^(52)^. DL models can also automatically classify submucosal tumors in esophageal EUS images while identifying invasion depth and lesion origin ^(53)^. For prostate cancer, a two-dimensional U-Net model achieved accurate segmentation of the clinical target volume corresponding to the prostate on 3D transrectal US images with brachytherapy-implanted needles ^(54)^. The well-trained EfficientNet B4 model was used to evaluate benign or malignant US skin tumor images and was shown to be at least as accurate as an experienced dermatologist in skin US diagnosis ^(55)^. Additionally, using 1,102 transvaginal US images from 796 patients with cervical cancer, U-Net-based automatic segmentation achieved high accuracy in delineating cervical cancer target areas ^(56)^. Furthermore, convolutional neural network -based DL models achieved an AUC of 0.85 for diagnosing rectal cancer using endoanal US ^(57)^.

## 3. Practical Issues to Be Addressed for the Clinical Application of AI to US Imaging

### 3.1. Domain shift

DL technology excels at image recognition and is extensively used in AI-SaMDs for medical image diagnosis. However, DL requires large amounts of training data, and clinical data are often limited. Collecting data from multiple facilities can lead to domain shifts, where the training and test data distributions do not match across different facilities or machines, resulting in reduced model performance. To address this, some solutions have been proposed, such as federated learning in a distributed dataset environment and domain adaptation and fine-tuning for multicenter and multimachine data using trained models ^(58), (59)^.

### 3.2. Explainability

**Figure 1.**
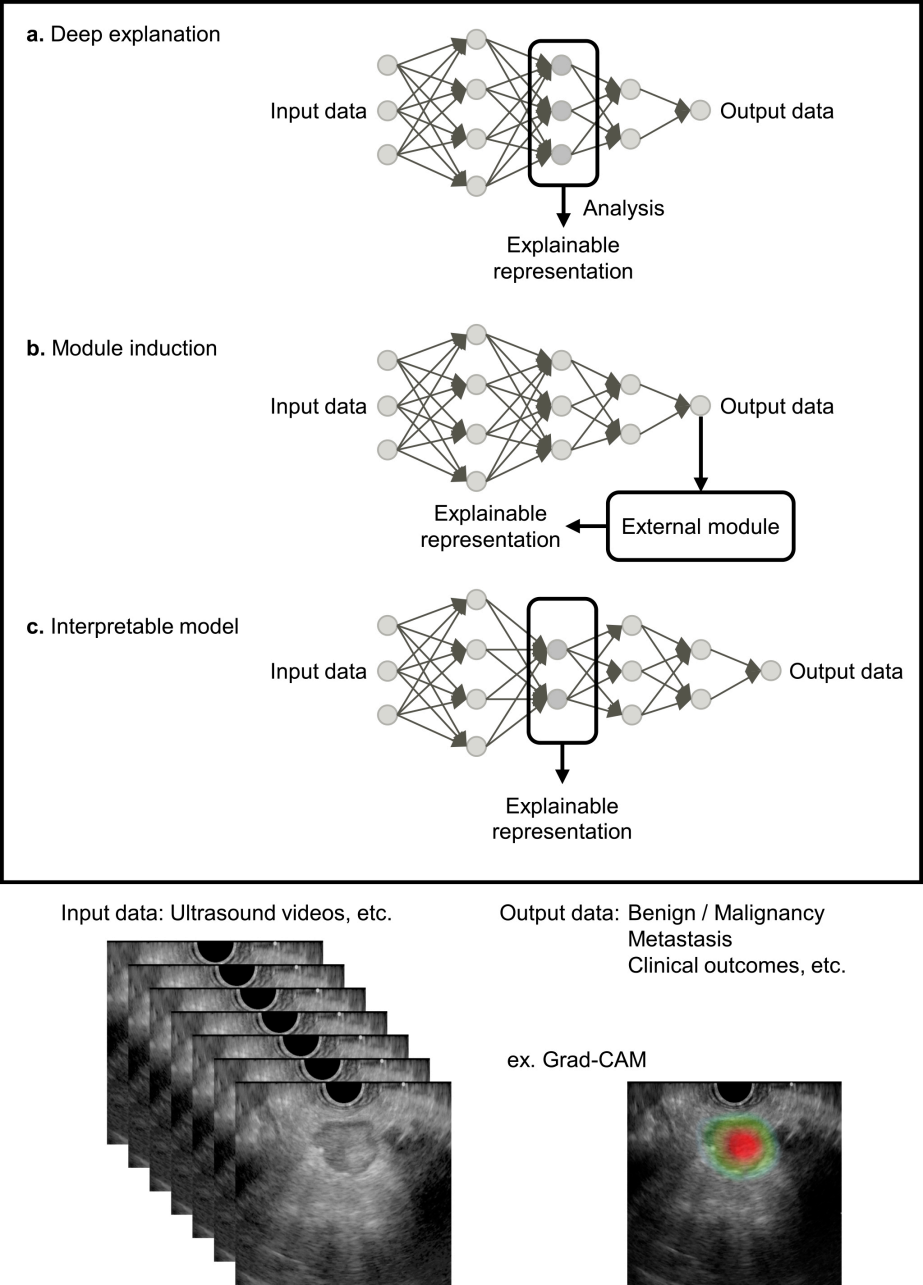
Approaches to explainable artificial intelligence (AI). (a) Deep explanation, (b) module induction, and (c) interpretable model have been proposed to generate human-understandable explanations of AI decisions. Grad-CAM, gradient-weighted class activation mapping.

The black box problem arises because humans cannot fully understand the decision-making process of DL models with complex convolutional neural network structures. Improving AI explainability is essential for medical professionals who use AI-based medical devices and for patients to provide informed consent based on diagnostic results ^(60)^. Countermeasures for the black box problem include visualizing the internal behavior of the model (deep explanation), adding an external explanation module to the model (module induction), and using other explainable representations (interpretable model) (Figure 1) ^(61), (62)^. A representative deep explanation method is a heat map display using gradient-weighted class activation mapping (Grad-CAM) ^(63)^. Grad-CAM uses the gradients of any target concept in the final convolutional layer to produce a coarse localization map that highlights important regions in the US image. Examples of interpretable models include barcodes and graph charts that display target area detection results in US videos ^(9), (11)^.

### 3.3. Acoustic shadow

**Figure 2.**
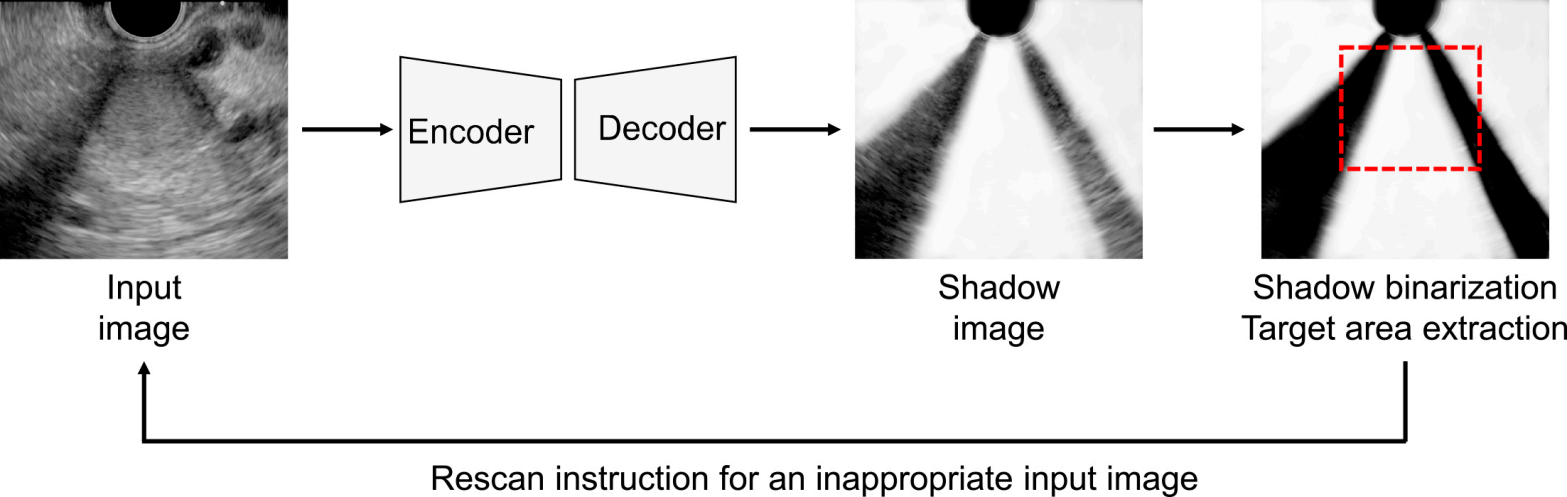
Automated detection and assessment of acoustic shadows in ultrasound (US) imaging. This encoder-decoder model detects acoustic shadows in US images and assesses their effect on the target area. When the model detects that an image is unsuitable for analysis due to acoustic shadows, it prompts the examiner to rescan the image.

Acoustic shadows are common artifacts that hinder the diagnosis of target organs in US and AI-based image recognition. Conventional AI-based methods for detecting acoustic shadows involve labor-intensive pixel-level annotation of semitransparent, blurred boundaries. To streamline this process, weakly supervised learning methods have been developed that use artificial shadows superimposed on US images as pseudo-labels for training models ^(64)^. Chen et al. proposed a semisupervised shadow-aware network with boundary refinement, which enhances the accuracy of shadow detection ^(65)^. These methods improve anomaly detection by automatically evaluating the effect of acoustic shadows, thereby guiding examiners to rescan problematic images (Figure 2). Additionally, a vendor-specific approach uses an ML model trained with preimaging data to differentiate between diagnostic noise and useful speckle signals, effectively filtering out unnecessary noise in US images.

## 4. Conclusions

In this review, we examined AI-based advances in US imaging within the field of oncology. Most studies have focused on tumor detection, differentiation between benign and malignant lesions, and prediction of lymph node metastasis, which aligns with clinical needs. Despite these advances, clinical implementation remains limited. The market for AI/ML-enabled medical devices is expected to grow due to increasing innovation. Future applications of AI in medicine are anticipated to enhance operational efficiency and support the development of infrastructures such as remote healthcare and digital medical databases. To successfully integrate AI into clinical US imaging for oncology, it is crucial to address and resolve practical challenges such as domain shifts, explainability, and acoustic shadows.

## Article Information

### Conflicts of Interest

None

### Sources of Funding

This work was supported by the Cabinet Office BRIDGE (programs for bridging the gap between R&D and the ideal society (Society 5.0) and generating economic and social value) and the MEXT subsidy for the Advanced Integrated Intelligence Platform.

### Acknowledgement

We thank all the members of the Hamamoto Laboratory for their valuable discussions.

### Approval by Institutional Review Board (IRB)

Not applicable.
